# Association Between Handover of Anesthesiology Care and 1-Year Mortality Among Adults Undergoing Cardiac Surgery

**DOI:** 10.1001/jamanetworkopen.2021.48161

**Published:** 2022-02-11

**Authors:** Louise Y. Sun, Philip M. Jones, Duminda N. Wijeysundera, Mamas A. Mamas, Anan Bader Eddeen, John O’Connor

**Affiliations:** 1 Division of Cardiac Anesthesiology, University of Ottawa Heart Institute, Ottawa, Ontario, Canada; 2 ICES, Ontario, Canada; 3 School of Epidemiology and Public Health, University of Ottawa, Ottawa, Ontario, Canada; 4 Departments of Anesthesia and Perioperative Medicine and Epidemiology and Biostatistics, University of Western Ontario, London, Ontario, Canada; 5 Department of Anesthesiology and Pain Medicine, University of Toronto, Toronto, Ontario, Canada; 6 Department of Anesthesia, St Michael’s Hospital, Toronto, Ontario, Canada; 7 Keele Cardiovascular Research Group, Centre for Prognosis Research, Keele University and Institute for Population Health, University of Manchester, United Kingdom

## Abstract

**Importance:**

Handovers of anesthesia care from one anesthesiologist to another is an important intraoperative event. Despite its association with adverse events after noncardiac surgery, its impact in the context of cardiac surgery remains unclear.

**Objective:**

To compare the outcomes of patients who were exposed to anesthesia handover vs those who were unexposed to anesthesia handover during cardiac surgery.

**Design, Setting, and Participants:**

This retrospective cohort study in Ontario, Canada, included Ontario residents who were 18 years or older and had undergone coronary artery bypass grafting or aortic, mitral, tricuspid valve, or thoracic aorta surgical procedures between 2008 and 2019. Exclusion criteria were non-Ontario residency status and other concomitant procedures. Statistical analysis was conducted from April 2021 to June 2021, and data collection occurred between November 2020 to January 2021.

**Exposures:**

Complete handover of anesthesia care, where the case is completed by the replacement anesthesiologist.

**Main Outcomes and Measures:**

The coprimary outcomes were mortality within 30 days and 1 year after surgery. Secondary outcomes were patient-defined adverse cardiac and noncardiac events (PACE), intensive care unit (ICU), and hospital lengths of stay (LOS). Inverse probability of treatment weighting based on the propensity score was used to estimate adjusted effect measures. Mortality was assessed using a Cox proportional hazard model, PACE using a cause-specific hazard model with death as a competing risk, and LOS using Poisson regression.

**Results:**

Of the 102 156 patients in the cohort, 25 207 (24.7%) were women; the mean (SD) age was 66.4 (10.8) years; and 72 843 of surgical procedures (71.3%) were performed in teaching hospitals. Handover occurred in 1926 patients (1.9%) and was associated with higher risks of 30-day mortality (hazard ratio [HR], 1.89; 95% CI, 1.41-2.54) and 1-year mortality (HR, 1.66; 95% CI, 1.31-2.12), as well as longer ICU (risk ratio [RR], 1.43; 95% CI, 1.22-1.68) and hospital (RR, 1.17; 95% CI, 1.06-1.28) LOS. There was no statistically significant association between handover and PACE (30 days: HR 1.09; 95% CI, 0.79-1.49; 1 year: HR 0.89; 95% CI, 0.70-1.13).

**Conclusions and Relevance:**

Handover of anesthesia care during cardiac surgical procedures was associated with higher 30-day and 1-year mortality rates and increased health care resource use. Further research is needed to evaluate and systematically improve the handover process qualitatively.

## Key Points

**Question:**

Is intraoperative handover of anesthesia care from one anesthesiologist to another associated with adverse outcomes perioperatively and at 1 year in patients who have cardiac surgical procedures?

**Findings:**

In this cohort study of 102 156 patients from Ontario, Canada, who were 18 years or older and had cardiac surgical procedures between 2008 and 2019, anesthesia handover was associated with a higher risk of death at 30 days and 1-year and with an increase in health care resource use.

**Meaning:**

These findings suggest that strategies are needed to balance the anesthesia clinician well-being and the adverse impact of physician fatigue with unintended information loss during the handover process.

## Teaser

This cohort study examines the outcomes of patients who were exposed to anesthesia handover vs those who were unexposed to anesthesia handover during cardiac surgery in Ontario, Canada, between 2008 and 2019.

## Introduction

The handover of care from one anesthesiologist to another is an important intraoperative event and a particularly vulnerable time for patients.^1,2,3^ Handovers are frequent in modern anesthesiology practice to prevent physician burnout by allowing predictability in daily work schedules and mitigate the adverse impact of clinician fatigue on patient care. It is frequent in the setting of cardiac surgical procedures and occurs in up to 6.7% of these cases compared with 3.5% of neurosurgical procedures and 0.8% of lung resections.^2,3,4^ Successful handover involves continuing provision of care by the primary anesthesiologist while effectively communicating key patient and procedure-related details to the replacement anesthesiologist.^5,6^ Conversely, patient safety could be compromised and continuity of care disrupted if key details are missed.

There are 2 types of anesthesiology handover. Temporary handover refers to the temporary absence of the primary anesthesiologist, who subsequently returns to complete the case. Complete handover occurs when the replacement anesthesiologist completes the case.^3^ Complete anesthesia handover has been implicated in perioperative mortality and adverse events in the setting of noncardiac surgery,^3^ but its impact on patients who undergo cardiac surgery remains unclear. In this population-based, multicenter study, we hypothesized that complete intraoperative anesthesia handover was associated with higher mortality rates, patient-defined adverse cardiovascular and noncardiovascular events (PACE),^7^ and health care resource use within 1 year of surgery.

## Methods

This population-based, retrospective cohort study followed the Strengthening the Reporting of Observational Studies in Epidemiology (STROBE) reporting guideline. The data set from this study is held securely in coded form at ICES (formerly the Institute for Clinical Evaluative Sciences). ICES is an independent, nonprofit research institute whose legal status under Ontario’s health information privacy law allows it to collect and analyze health care and demographic data, without consent, for health system evaluation and improvement. The use of data was authorized under section 45 of Ontario’s *Personal Health Information Protection Act*, which does not require review by a research ethics board.^8^

### Study Design, Setting, and Population

This study included adult patients aged 18 years or older who had coronary artery bypass grafting (CABG) or aortic, mitral, tricuspid valve, or thoracic aorta surgical procedures in Ontario, Canada, between October 1, 2008, and September 30, 2019. Only the first procedure was included in the analyses for patients with multiple eligible procedures during the study period. Exclusion criteria were having non-Ontario residency status, missing information regarding age and sex, and having other concomitant procedures (eFigure in the Supplement). During the study period, Ontario was Canada’s most populous province with a publicly funded, universal health care system that reimbursed all covered services and clinicians.

### Data Sources

We used the detailed clinical registry data from CorHealth Ontario and ICES administrative health care databases with information about all Ontario residents. ICES is an independent, nonprofit research institute whose legal status under Ontario’s health information privacy law allows it to collect and analyze health care and demographic data, without consent, for health system evaluation and improvement. CorHealth Ontario maintains a detailed prospective registry of all patients who undergo invasive cardiac diagnostic and therapeutic procedures in Ontario, including demographic, comorbidity, and procedure-related information. CorHealth Ontario data were prospectively collected from the time of surgical referral and underwent selected chart audits and core laboratory validation.^8,9,10,11,12,13,14,15,16,17,18^

All analyses were performed at ICES by using unique confidential identifiers to deterministically link the CorHealth registry (date and type of cardiac procedures, physiologic and comorbidity data) with the Canadian Institute for Health Information Discharge Abstract Database (DAD; comorbidities and hospital admissions) and Same Day Surgery (SDS) database (comorbidities), the Ontario Health Insurance Plan (OHIP) database (physician service claims), the Registered Persons Database (RPDB; vital statistics), Continuing Care Reporting System (CCRS; admissions to long-term care facilities), Assistive Devices Program (ADP; ventilator supplies), Canadian Organ Replacement Registry (CORR), National Rehabilitation Reporting System (NRS; rehabilitation), and ICES Physician Database (IPDB) and Canadian Physician Database (CPDB) (physician demographics). These administrative databases have been validated for many outcomes, exposures, and comorbidities, including heart failure, chronic obstructive pulmonary disease, asthma, hypertension, myocardial infarction, and diabetes.^19,20,21,22^

### Exposure

Complete handover of anesthesia care, where the case is completed by the replacement anesthesiologist, was identified from OHIP using the billing code E005C submitted by the replacement anesthesiologist within 1 day of surgery. This method is expected to be accurate because it is the only means to remunerate the replacement anesthesiologist in Ontario and has been previously used to study anesthesia handover in noncardiac surgery.^3^

### Outcomes

The coprimary outcomes were all-cause mortality within 30 days and 1 year after surgery. Secondary outcomes were postoperative length of stay (LOS) in the hospital and the intensive care unit (ICU) during the index surgical encounter and PACE within 30 days and up to 1 year after surgery. PACE is a patient-centered outcome codeveloped by cardiac patients, caregivers, and clinicians.^7^ It comprises severe stroke that leads to hospitalization of 14 days or longer or inpatient rehabilitation (DAD, NRS),^15,23^ chronic ventilator dependence (ADP and OHIP), new-onset HF (DAD, SDS, NACRS, OHIP, and OMHRS),^24^ new-onset dialysis (DAD, SDS, OHIP, and CORR) and long-term care admission (CCRS).^7,15,23^

### Covariates

Covariates considered in the analyses are listed in Table 1 and included patient demographic and comorbidities, operative factors including procedure type and duration, hospital, anesthesiologist, and surgeon characteristics. Like our previous studies,^8,9,10,11,12,13,14,15,16,17,25,26,27,28^ height, weight, operative priority, left ventricular ejection fraction, and valvular heart disease were identified from the CorHealth registry. Other comorbidities were identified from CorHealth and supplemented with data from DAD, SDS, and OHIP using *International Statistical Classification of Diseases and Related Health Problems, Tenth Revision* (*ICD-10*) codes^29^ within 5 years before the index procedure, according to established algorithms.^19,21,24,30^ Clinician characteristics were obtained from the IPDB and CPDB.

**Table 1.** Baseline Characteristics of Patients Before and After Inverse Probability of Treatment Weighting

Characteristic	Observed data (n = 102 156)			IPTW data (n = 204 531)		
	No. (%)		ASD	No. (%)		ASD
	Handover (n = 1926)	No handover (n = 100 230)		Handover (n = 102 376.2)	No handover (n = 102 155.8)	
Demographics						
Age, mean (SD), y	64.8 (11.5)	66.4 (10.8)	0.15	66.8 (10.8)	66.4 (10.8)	0.04
Female	435 (22.6)	24 772 (24.7)	0.05	23 038 (22.5)	25 209 (24.7)	0.05
Male	1491 (77.4)	75 458 (75.3)	0.05	79 338.2 (77.5)	79 946.8 (75.3)	0.05
BMI, mean (SD)	28.9 (7.5)	29 (7)	0.01	29.3 (8.3)	29 (6.9)	0.04
Rural residence	262 (13.6)	15 318 (15.3)	0.05	19 199.6 (18.8)	15 583 (15.3)	0.09
Hospital type						0.00
Community	227 (11.8)	29 086 (29.0)	0.44	29 468.8 (28.8)	29 313.2 (28.7)	0.00
Teaching	1699 (88.2)	71 144 (71.0)	0.44	72 907.3 (71.2)	72 842.5 (71.3)	0.00
Income quintile						
1	432 (22.4)	19 099 (19.1)	0.08	18 312.1 (17.9)	19 528.8 (19.1)	0.03
2	345 (17.9)	20 505 (20.5)	0.06	19 186.8 (18.7)	20 851.3 (20.4)	0.04
3	412 (21.4)	20 568 (20.5)	0.02	22 105.4 (21.6)	20 979.4 (20.5)	0.03
4	381 (19.8)	20 237 (20.2)	0.01	22 265.7 (21.7)	20 620 (20.2)	0.04
5	356 (18.5)	19 821 (19.8)	0.03	20 506.2 (20.0)	20 176.3 (19.8)	0.01
Comorbidities						
Hypertension	1592 (82.7)	85 417 (85.2)	0.07	88 095 (86.1)	87 009.6 (85.2)	0.02
Atrial fibrillation	127 (6.6)	6978 (7.0)	0.01	7470.8 (7.3)	7103.6 (7.0)	0.01
Recent MI	497 (25.8)	23 010 (23.0)	0.07	26 308.4 (25.7)	23 507.3 (23.0)	0.06
CCS class						
0	342 (17.8)	21 685 (21.6)	0.10	19 444.4 (19.0)	22 026.3 (21.6)	0.06
1	208 (10.8)	9453 (9.4)	0.05	8193.6 (8.0)	9660.2 (9.5)	0.05
2	260 (13.5)	16 458 (16.4)	0.08	16 026.6 (15.7)	16 717.4 (16.4)	0.02
3	165 (8.6)	14 705 (14.7)	0.19	15 840.3 (15.5)	14 869.6 (14.6)	0.03
4	138 (7.2)	3442 (3.4)	0.17	2657.5 (2.6)	3578.4 (3.5)	0.05
ACS						
Low risk	201 (10.4)	15 090 (15.1)	0.14	16 707.2 (16.3)	15 291.6 (15.0)	0.04
Intermediate risk	258 (13.4)	13 018 (13.0)	0.01	16 220.9 (15.8)	13 278.2 (13.0)	0.08
High risk	153 (7.9)	3788 (3.8)	0.18	3841.2 (3.8)	3941.6 (3.9)	0.01
Emergent	201 (10.4)	2591 (2.6)	0.32	3444.5 (3.4)	2792.5 (2.7)	0.04
PAD	254 (13.2)	13 439 (13.4)	0.01	13 216.1 (12.9)	13 692.3 (13.4)	0.01
LVEF						
≥50%	1378 (71.5)	70 641 (70.5)	0.02	70 631.4 (69.0)	72 018.4 (70.5)	0.03
35%-49%	375 (19.5)	20 462 (20.4)	0.02	22 638.4 (22.1)	20 836.1 (20.4)	0.04
20%-35%	149 (7.7)	7755 (7.7)	0.00	7804.8 (7.6)	7905.2 (7.7)	0.00
<20%	24 (1.2)	1372 (1.4)	0.01	1301.6 (1.3)	1396.1 (1.4)	0.01
NYHA class						
1	824 (42.8)	44 961 (44.9)	0.04	47 545.1 (46.4)	45 786.8 (44.8)	0.03
2	197 (10.2)	15 057 (15.0)	0.14	16 226.1 (15.8)	15 253.7 (14.9)	0.03
3	136 (7.1)	12 138 (12.1)	0.17	11 386.6 (11.1)	12 273.5 (12.0)	0.03
4	126 (6.5)	2815 (2.8)	0.18	2526.2 (2.5)	2940.5 (2.9)	0.03
Heart failure	502 (26.1)	26 699 (26.6)	0.01	30 513.7 (29.8)	27 203.1 (26.6)	0.07
Endocarditis						
None	1881 (97.7)	98 861 (98.6)	0.07	100 771.9 (98.4)	100 741.2 (98.6)	0.02
Active	35 (1.8)	981 (1.0)	0.07	1186.3 (1.2)	1016.3 (1.0)	0.02
Subacute	10 (0.5)	388 (0.4)	0.02	418 (0.4)	398.3 (0.4)	0.00
Cerebrovascular disease	165 (8.6)	9843 (9.8)	0.04	9771.2 (9.5)	10 005.7 (9.8)	0.01
Smoker						
Never	952 (49.4)	46 445 (46.3)	0.06	46 545.3 (45.5)	47 399.5 (46.4)	0.02
Current	352 (18.3)	19 378 (19.3)	0.03	19 310.7 (18.9)	19 729 (19.3)	0.01
Former	622 (32.3)	34 407 (34.3)	0.04	36 520.1 (35.7)	35 027.3 (34.3)	0.03
Diabetes	512 (26.6)	29 261 (29.2)	0.06	28 843.7 (28.2)	29 772.1 (29.1)	0.02
GFR, mL/min/1.73 m^2^	74.8 (22.6)	73.7 (21.6)	0.05	73.1 (21.5)	73.7 (21.6)	0.03
Dialysis	57 (3.0)	2136 (2.1)	0.05	2572.2 (2.5)	2192.6 (2.1)	0.02
Anemia	291 (15.1)	10 263 (10.2)	0.15	9542.7 (9.3)	10 552.5 (10.3)	0.03
Liver disease	20 (1.0)	979 (1.0)	0.01	1032.7 (1.0)	999.2 (1.0)	0.00
Dementia	23 (1.2)	1315 (1.3)	0.01	1439.6 (1.4)	1337.7 (1.3)	0.01
Depression	26 (1.3)	1413 (1.4)	0.01	1459.1 (1.4)	1439.4 (1.4)	0.00
Psychosis	1-5^a^	203-207^a^	0.01	153.6-204.8^a^	153.2-204.3^a^	0.01
Malignant neoplasm	78 (4.0)	5129 (5.1)	0.05	4730.3 (4.6)	5206.6 (5.1)	0.02
Operative characteristics						
Surgery type						
CABG	1155 (60.0)	65 449 (65.3)	0.11	69 794 (68.2)	66 603.8 (65.2)	0.06
Multi valve	30 (1.6)	1791 (1.8)	0.02	1586.8 (1.5)	1821.4 (1.8)	0.02
Single valve	209 (10.9)	14 678 (14.6)	0.11	11 991.3 (11.7)	14 884.7 (14.6)	0.08
CABG and Single valve	149 (7.7)	9973 (10.0)	0.08	10 620.9 (10.4)	10 122.4 (9.9)	0.02
CABG and Multivalve	14 (0.7)	721 (0.7)	0.00	690.4 (0.7)	735.1 (0.7)	0.01
Thoracic aorta	369 (19.2)	7618 (7.6)	0.34	7692.8 (7.5)	7988.4 (7.8)	0.01
Redo sternotomy	75 (3.9)	2779 (2.8)	0.06	3517.4 (3.4)	2854.1 (2.8)	0.04
Cardiogenic shock	34 (1.8)	438 (0.4)	0.13	441.2 (0.4)	471.2 (0.5)	0.00
Operative priority						
Emergent	286 (14.8)	5241 (5.2)	0.32	5842 (5.7)	5526.8 (5.4)	0.01
Urgent	568 (29.5)	30 884 (30.8)	0.03	33 092 (32.3)	31 454.1 (30.8)	0.03
Semiurgent	476 (24.7)	26 312 (26.3)	0.04	25 279.8 (24.7)	26 786.2 (26.2)	0.04
Elective	596 (30.9)	37 793 (37.7)	0.14	38 162.3 (37.3)	38 388.6 (37.6)	0.01
Physician characteristics						
Surgeon						
Age, mean (SD), y	49.5 (8.4)	49.9 (8.9)	0.05	49.5 (8.3)	49.9 (8.9)	0.05
Female	111 (5.8)	8631 (8.6)	0.11	8927.3 (8.7)	8739.9 (8.6)	0.01
Male	1815 (94.2)	91 599 (91.4)	0.11	93 448.9 (91.3)	93 415.9 (91.4)	0.01
Volume, mean (SD)^b^	2629.3 (1908.7)	2853 (1852.1)	0.12	2700.2 (2023.9)	2848.5 (1851.2)	0.08
Anesthesiologist						
Age, mean (SD), y	48.6 (9.1)	48.3 (9)	0.03	47.9 (9)	48.3 (9)	0.04
Female	494 (25.6)	19 587 (19.5)	0.15	17 888.8 (17.5)	20 078.7 (19.7)	0.06
Male	1432 (74.4)	80 643 (80.5)	0.15	84 487.3 (82.5)	82 077.1 (80.3)	0.06
Volume^b^						
<500	628 (32.6)	34 783 (34.7)	0.04	40 049.7 (39.1)	35 414.4 (34.7)	0.09
500-999	568 (29.5)	27 012 (27.0)	0.06	29 489.5 (28.8)	27 580.7 (27.0)	0.04
1000-1999	620 (32.2)	32 708 (32.6)	0.01	28 280 (27.6)	33 325.1 (32.6)	0.11
≥2000	110 (5.7)	5727 (5.7)	0.00	4556.9 (4.5)	5835.5 (5.7)	0.06
Surgery duration, min						
<300	713 (37.0)	66 694 (66.5)	0.62	66 421.1 (64.9)	67 406.1 (66.0)	0.02
300-479	957 (49.7)	31 191 (31.1)	0.39	33 121.2 (32.4)	32 148.2 (31.5)	0.02
≥480	256 (13.3)	2345 (2.3)	0.42	2833.9 (2.8)	2601.4 (2.5)	0.01

Abbreviations: ACS, acute coronary syndrome; ASD, absolute standardized difference; BMI, body mass index calculated as weight in kilograms divided by height in meters squared; CABG, coronary artery bypass grafting; CCS, Canadian Cardiovascular Society; GFR, glomerular filtration rate; IPTW, inverse probability of treatment weighting; LVEF, left ventricle ejection fraction; MI, myocardial infarction; NYHA, New York Heart Association; PAD, peripheral arterial disease.

^a^ Numbers suppressed due to small cells.

^b^ Surgeon and anesthesiologist volume is defined as total number of cardiac surgical procedures performed since April 1, 1991.

### Statistical Analysis

Where appropriate, continuous variables were compared across exposure status with 2-sample *t* test or Wilcoxon rank sum test. Categorical variables were compared with a χ^2^ test. We assessed mortality through September 30, 2020, and PACE through March 31, 2020. Patients were censored when they lost possession of a valid Ontario health insurance card. Event time was defined as the date of the index surgical procedure until the date of the event or the date of the last follow-up, whichever occurred earlier.

To account for the differences in characteristics between patients who were exposed to anesthesia handover vs not exposed to anesthesia handover, we used inverse probability of treatment weighting (IPTW) based on propensity scores to estimate the effect of the anesthesiologist handover while controlling for baseline patient, procedure, hospital, and clinician characteristics between groups. Specifically, we used logistic regression to estimate the propensity scores of anesthesiology handover, using a priori selected variables (Table 1). Subsequent analyses were performed in the sample weighted by the inverse probability of handover, within the common support region with a lower bound defined by the maximum of the minimum propensity score in the exposed and nonexposed groups and the upper bound defined by the minimum of the maximum propensity score in the 2 groups.^32^ Mortality rates in each group were calculated using the Kaplan-Meier method, and the impact of handover on mortality was estimated using Cox proportional hazard regression with robust standard errors to account for clustering at the patient level. We estimated the incidence of PACE over time using cumulative incidence functions (CIFs), and the impact of handover on PACE using a cause-specific hazard model, with death as a competing risk. We modeled ICU and hospital LOS using the Poisson regression.

#### Subgroup and Exploratory Analyses

We conducted prespecified subgroup analyses by stratifying the inverse probability of the treatment–weighted cohort according to: (1) simple (isolated CABG, single valve) vs complex surgical procedures (CABG and valve, multiple valves, thoracic aorta) and (2) daytime vs evening, night, or weekend case start.

We conducted an exploratory analysis by comparing the outcomes of patients whose handover occurred before cardiopulmonary bypass, during and after bypass, and without anesthesia handover. Because the precise timing of the bypass is not readily available in the database we used, we designated handover during and after bypass as occurring during the last two-thirds of the recorded anesthesia duration.

Analyses were performed using SAS 9.4 (SAS Institute), with statistical significance defined by a 2-sided *P* value of <.05. Measures of association were hazard ratios (HR) for binary outcomes and rate ratios (RR) for continuous outcomes, with 95% CIs.

#### Sensitivity Analyses

We conducted 2 sensitivity analyses to test the robustness of our findings. First, we used negative binomial regression to estimate the association between handover and LOS in the IPTW cohort. Second, we modeled the association between handover and each outcome using multivariable Cox proportional hazard regression for mortality, cause-specific hazard regression for PACE, and Poisson and negative binomial regression for ICU and hospital LOS. In addition to covariates used in the propensity score, we also adjusted for the year of the surgical procedure and accounted for clustering at the anesthesiologist’s level in each of these multivariable models.

#### Missing Data

Left ventricle ejection fraction was missing in 3497 patients (3.4%), glomerular filtration rate in 4510 (4.4%), operative priority in 11 683 (11.4%), income in 263 (0.3%), rurality in 84 (0.1%), and surgery duration in 951 (0.9%) patients. These missing values were imputed within the SAS proc MI framework, where they were estimated drawing on all candidate covariates using the predictive mean matching imputation method for continuous variables and logistic regression for categorical variables.^31^

## Results

**Figure 1.**
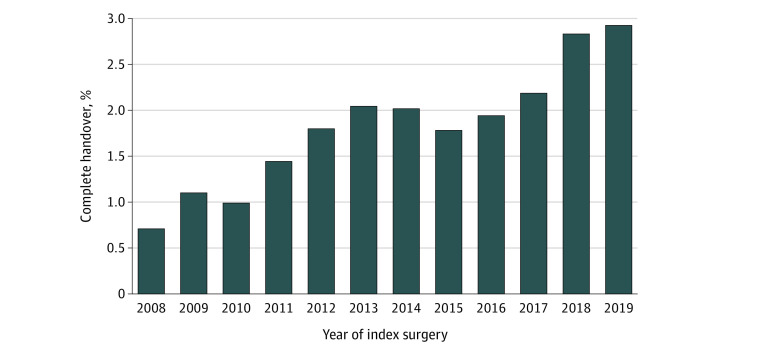
Temporal Trend in the Proportion of Complete Anesthesia Handover in Ontario

Of a total of 102 156 patients who met the inclusion criteria and fell within the area of common support after IPTW, 25 207 (24.7%) were women; the mean (SD) age was 66.4 (10.8) years; 72 843 (71.3%) of the surgical procedures were performed in teaching hospitals; 38 389 (37.6%) of the surgical procedures were elective; 20 081 (19.7%) of primary anesthesiologists were women. A total of 1926 patients (1.9%) experienced complete handover of anesthesia care. The rate of anesthesia handover has increased during the study period, from 0.7% in 2008 to 2.9% in 2019 (Figure 1). Several important differences in baseline characteristics were noted between groups with complete handover and no handover (Table 1). Specifically, compared with those without handover, patients in the handover group were more likely to be treated at teaching hospitals (71 144 of 100 230 [71.0%] vs 1699 of 1926 [88.2%]); to have prolonged surgery duration of 300 minutes or longer (33 536 of 100 230 [33.4%] vs 1213 of 1926 [63%]); to undergo emergent surgical procedures (5241 of 100 230 [5.2%] vs 286 of 1926 [14.8%]); thoracic aorta surgical procedures (7618 of 100 230 [7.6%] vs 369 of 1926 [19.2%]); to present with cardiogenic shock (438 of 100 230 [0.4%] vs 34 of 1926 [1.8%]), more severe anginal and HF symptoms (18 147 of 102 230 [18.1%] had CCS Class 3-4 vs 303 of 1926 [15.7%]; 27 195 of 102  230 [27.1%] had NYHA Class 2-3 vs 333 of 1926 [17.3%]), to be treated by less experienced surgeons (mean [SD] surgeon procedure volume, 2629.3 [1908.7] vs 2853 [1852.1]), and to have female primary anesthesiologists (19 587 of 100 230 [19.5%] vs 494 of 1926 [25.6%]). The groups were clinically well balanced after IPTW.

### Adjusted Outcomes

**Figure 2.**
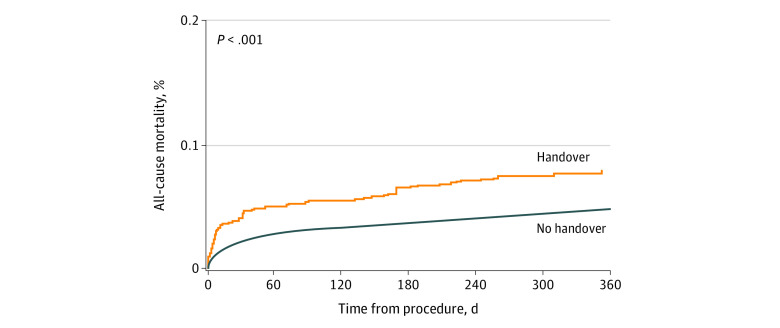
Estimated 1-Year Mortality in the Inverse Probability of Treatment–Weighted Cohort

**Figure 3.**
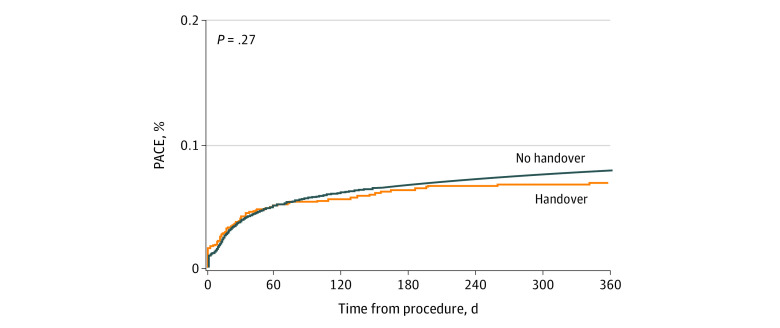
Cumulative Incidence of Patient-Defined Adverse Cardiovascular and Noncardiovascular Events (PACE) in the Inverse Probability of Treatment Weighted Cohort

The 30-day and 1-year outcomes of patients with and without anesthesia handover in the inverse probability of treatment–weighted cohort are summarized in Table 2. The estimated 1-year survival and PACE CIF curves are presented in Figure 2 and Figure 3, respectively. Handover was associated with a higher risk of mortality at 30 days (HR, 1.89; 95% CI, 1.41-2.54; population attributable risk [PAR], 0.017; 38 deaths were attributable to handover at 30-day) and at 1-year (HR, 1.66; 95% CI, 1.31-2.12; PAR, 0.012; 62 deaths were attributable to handover at 1-year), as well as prolonged ICU (RR, 1.43; 95% CI, 1.22-1.68) and hospital LOS (RR, 1.17; 95% CI, 1.06-1.28). There was no statistically significant association between handover and PACE in the weighted cohort (HR 1.09; 95% CI, 0.79-1.49; PAR, 0.0017; 7 PACE events were attributable to handover at 30 days; HR, 0.89; 95% CI, 0.70-1.13; PAR, −0.0021; −17 PACE events were attributable to handover at 1-year).

**Table 2.** Main Outcomes in the Original and Inverse Probability of Treatment–Weighted Cohort

Outcome	No. (%)		Effect measure (95% CI)^a^	*P* value
	Handover	No handover		
**Observed data **				
No. (n = 102 156)	1926	100 230		
Primary outcome				
All-cause death				
Within 30 d	133 (6.9)	2173 (2.2)	1.50 (1.25-1.81)	<.001
Within 1 y	200 (10.4)	4841 (4.8)	1.52 (1.31-1.76)	<.001
Secondary outcomes				
PACE				
Within 30 d	102 (5.3)	4001 (4.0)	1.13 (0.92-1.39)	.23
Within 1 y	156 (8.1)	7987 (8.0)	1.00 (0.85-1.18)	.99
LOS, median (IQR), d				
ICU	2 (1-5)	1 (1-3)	1.32 (1.22-1.41)	<.001
Hospital	7 (5-11)	6 (5-8)	1.16 (1.09-1.23)	<.001
**IPTW data **				
No. (n = 204 531)	102 376.2	102 155.8		
Primary outcome				
All-cause death				
Within 30 d	4261 (4.2)	2269.8 (2.2)	1.89 (1.41-2.54)	<.001
Within 1 y	8173.8 (8.0)	4995.3 (4.9)	1.66 (1.31-2.12)	<.001
Secondary outcomes				
PACE				
Within 30 d	4383.3 (4.3)	4093.9 (4.0)	1.09 (0.79-1.49)	.61
Within 1 y	7118.4 (7.0)	8154.8 (8.0)	0.89 (0.70-1.13)	.35
LOS, median (IQR), d				
ICU	2 (1-3)	1 (1-3)	1.43 (1.22-1.68)	<.001
Hospital	6 (5-9)	6 (5-8)	1.17 (1.06-1.28)	.001

Abbreviations: ICU, intensive care unit; IPTW, inverse probability of treatment weighting; LOS, length of stay; PACE, patient-relevant adverse cardiac and noncardiac events.

^a^ Hazard ratios were provided for binary outcomes (death, PACE) and rate ratios were provided for continuous outcomes (ICU and hospital LOS).

### Subgroup Analysis

The results from the prespecified subgroup analyses are summarized in eTable 1 in the Supplement. In the analysis stratified by procedural complexity, the association between handover and each outcome was greater in patients who underwent complex surgery. Moreover, procedure complexity modified the effect of anesthesia handover on 1-year mortality (HR, 2.21 [1.61-3.02]) and 30-day PACE (1.85 [1.17-2.93]), such that handover was associated with these outcomes only in patients undergoing complex surgical procedures. In the analysis stratified by the timing of surgery case start (daytime vs evening, night, or weekend), handover was associated with increased perioperative (HR 2.14 [1.54-2.95]) and 1-year mortality (HR, 1.65 [1.26-2.16]), ICU (RR, 1.35 [1.19-1.52]) and hospital LOS (RR, 1.12 [1.03-1.21]) only in cases started during regular workdays.

### Exploratory Analysis

In the exploratory analysis by the timing of handover relative to cardiopulmonary bypass, handover was associated with 30-day and 1-year mortality, ICU, and hospital LOS irrespective of its timing. However, handover during or after bypass had a stronger impact on 30-day mortality (HR 2.25 [1.45-3.50] vs 1.73 [1.18-2.54]) and is associated with PACE at 30 days (HR 1.77 [1.11-2.83]) (eTable 2 in the Supplement).

### Sensitivity Analysis

In the IPTW cohort, the association between handover and LOS remained robust in the analysis that used negative binomial regression (ICU LOS: RR, 1.41 [1.19-1.67] vs 1.43 [1.22-1.68]; hospital LOS: RR, 1.15 [1.05-1.27] vs 1.17 [1.06-1.28]). In the original cohort, the association between handover and each outcome remained robust when modeled using multivariable regression (Table 2; eTable 3 to eTable 10 in the Supplement).

## Discussion

In this multicenter, population-based study, we found a clinically and statistically significant association between anesthesiology handover and perioperative and 1-year mortality, as well as longer ICU and hospital LOS. This association persisted in our subgroup and exploratory analyses, where: (1) handover during complex procedures was associated with an exacerbation of all outcomes; (2) handover was associated with increased mortality and LOS only in cases started during regular work hours; and (3) patients whose care was transitioned during or after bypass had greater rates of mortality and PACE perioperatively. Novel aspects of our study include patient follow-up beyond the perioperative period in a large and representative patient population, inclusion of anesthesiologist and surgeon characteristics in our analyses, the performance of important subgroup analyses, and the addition of PACE as a patient-relevant outcome.

There is a paucity of studies to quantitatively describe the impact of anesthesiology handover on patient outcomes, with only 1 single-center study in the cardiac surgery setting. Hudson et al,^2^ in a historical cohort of 14 421 patients who underwent cardiac surgery between 1999 to 2009 at a tertiary center in Ontario, found that anesthesiology handover was associated with increased odds of in-hospital mortality (adjusted OR, 1.43; 95% CI, 1.01-2.01) as well as the LOS-prolonging composite endpoint of myocardial infarction, stroke, mechanical ventilation of more than 48 hours, and renal replacement therapy (adjusted OR, 1.27; 95% CI, 1.04-1.56). This single-center study is dated and has limited generalizability in the wider health care setting. Our study builds on these findings in a contemporary population-based cohort comprising patients from all 11 cardiac surgical procedure centers in the most populous province in Canada. We observed similar magnitudes of effect in our study that persisted 1-year after surgery and within subgroups by surgical complexity and handover timing.

A 2020 systematic review^1^ identified 6 quantitative studies on the potential impact of anesthesiology handover on more than 600 000 patients who underwent mostly noncardiac surgery. Of these studies, 5 demonstrated adverse outcomes associated with handover^2,3,33,34,35^ and 1 demonstrated a lack of association.^34,36^ In their exploratory meta-analysis of 3 noncardiac studies^3,33^ and 1 cardiac study,^2^ the authors reported a pooled relative risk of 1.40 (95% CI, 1.19-1.65; I^2^ = 98%) for mortality in patients who were exposed as compared to those not exposed to handover. Of note, the meta-analysis results were driven by a recent population-based study of 313 066 noncardiac surgery patients in Ontario.^3^ In this study, complete anesthesiology handover was associated with absolute risks of 1.2% (95% CI, 0.5%-2.0%) for 30-day mortality, 5.8% (3.6%-7.9%) for major complications, and 1.2% (95% CI, 0.7-1.7%) for hospital LOS. The systematic review also identified 2 qualitative studies that examined the process of anesthesiology handover from the perspective of anesthesiology clinicians. An online survey of 216 anesthesiologists in the US found patient complications or mismanagement because of poor handovers as a prevalent event experienced by 93% of respondents.^37^ A 1982 study^38^ that interviewed 91 US-based anesthesiologists reported that the relief anesthesiologist picked up 9% of preventable errors, and that the relief anesthesiologist discovered an error or the cause of an error in 29% of these cases.

The congruency of our findings with the majority of published quantitative research suggests that intraoperative anesthesiology handover during cardiac surgery is also associated with unintended harmful consequences that have clinically important ramifications far beyond the perioperative period. Handovers are increasingly frequent in modern medical practice because of policies of restricted duty hours to reduce physician fatigue, a known risk factor for reduced vigilance and preventable medical errors.^39,40^ Additional drivers of the need for the handover of anesthesia care include organizational efficiency to allow for the designation of on-call staff to take over longer cases and the need for predictable working hours to enhance physician well-being and prevent physician burnout. Around the world, approximately 2 million patients undergo cardiac surgery each year.^41^ Given the increasing prevalence of handover and its implication in adverse patient outcomes and resource use, research to identify at-risk patient-clinician combinations and strategies to improve the quality of communication is essential to safe health care delivery.

We identified several patient-, procedure-, and physician-related features of anesthesiology handover during cardiac surgery, which could be used to direct interventions to improve patient outcomes. These factors include teaching hospital; surgeon inexperience; female primary anesthesiologist; thoracic aorta surgery; and prolonged, emergent procedures in symptomatic and unstable patients. In addition, our subgroup and exploratory analysis revealed the added risk of handover during complex procedures, procedures started during regular working hours, and during and after cardiopulmonary bypass. Whereas structured handover from the operating room team to the receiving ICU team has been shown to enhance continuity of care, clinician satisfaction, and patient outcomes,^42,43^ intraoperative handover is often associated loss of important intangible information in the context of physician fatigue and often limited amount of time for the replacement anesthesiologist to get up to speed on key aspects of the procedure and to gain an intuitive grasp of the patient’s physiology. This highlights the need for anesthesiologists, surgeons, and administrators to codevelop and implement standardized electronic handover tools to improve patient safety in these high-risk situations. We have shown that separation from cardiopulmonary bypass is a particularly important event during which situational awareness could be lost. Therefore, caution is needed when handing over patient care during this period.

The strengths of this study include the large, representative study population, 1-year follow-up period, consideration of anesthesiologist and surgeon characteristics in the risk adjustment, as well as using PACE as a patient-relevant outcome that goes beyond the traditional clinician-centric measures.^7^ As the cohort involved only physician anesthesiologists, our findings are directly focused on the process of handover rather than the impact of different types of clinicians involved.

### Limitations

This study has several limitations. First, billing codes for replacement anesthesiologists more accurately identify handovers occurring in individual fee-for-service environments than revenue-sharing group academic practices where there is little financial incentive for using this billing code. This potential misclassification biases our results toward the null.^3^ Second, we were unable to ascertain the reason for the handovers, nor the presence of anesthesia trainees, based on the data sources available. Third, our analysis of handover timing relative to cardiopulmonary bypass was exploratory as the timing of bypass was not captured in our data sets. Finally, cohort studies are by nature subject to residual confounding.

## Conclusions

In this study, complete intraoperative handover of anesthesia care was associated with a higher risk of death at 30 days and 1-year and increased health care resource use compared with no handover. Further research is needed to devise strategies to balance the well-being of anesthesia clinicians and the adverse impact of physician fatigue with unintended information loss during the handover process.

## Notes

Supplement. **eTable 1.** Subgroup Analyses by Surgical Complexity and Timing of Handover in the Inverse Probability of Treatment Weighted Cohort **eTable 2.** Exploratory Analysis by the Timing of Anesthesia Handover Relative to Cardiopulmonary Bypass in the Inverse Probability of Treatment Weighted Cohort **eTable 3.** Sensitivity Analysis Where 30-Day Mortality Was Modeled Using Multivariable Cox Proportional Hazard Regression in the Original Cohort **eTable 4.** Sensitivity Analysis Where 1-Year Mortality Was Modeled Using Multivariable Cox Proportional Hazard Regression in the Original Cohort **eTable 5.** Sensitivity Analysis Where 30-Day PACE Was Modeled Using Multivariable Cause-Specific Hazard Regression in the Original Cohort **eTable 6.** Sensitivity Analysis Where 1-Year PACE Was Modeled Using Multivariable Cause-Specific Hazard Regression in the Original Cohort **eTable 7.** Sensitivity Analysis Where ICU Length of Stay Was Modeled Using Multivariable Poisson Regression in the Original Cohort **eTable 8.** Sensitivity Analysis Where ICU Length of Stay Was Modeled Using Multivariable Negative Binomial Regression in the Original Cohort **eTable 9.** Sensitivity Analysis Where Hospital Length of Stay Was Modeled Using Multivariable Poisson Regression in the Original Cohort **eTable 10.** Sensitivity Analysis Where Hospital Length of Stay Was Modeled Using Multivariable Negative Binomial Regression in the Original Cohort **eFigure.** Cohort Flow Diagram
