# Errors in Figure 2

**DOI:** 10.1001/jamanetworkopen.2024.14027

**Published:** 2024-04-24

In the Original Investigation titled “Association Between Handover of Anesthesiology Care and 1-Year Mortality Among Adults Undergoing Cardiac Surgery,”^1^ published February 11, 2022, the labels for the handover and no handover lines were switched in Figure 2. This article has been corrected.^1^
