# Supplementary material for: Association Between Handover of Anesthesiology Care and 1-Year Mortality Among Adults Undergoing Cardiac Surgery
Source: JAMA Netw Open. 2022 Feb 11;5(2):e2148161. doi: 10.1001/jamanetworkopen.2021.48161 (PMC8837916; doi:10.1001/jamanetworkopen.2021.48161)
Supplement: Supplement. — eTable 1. Subgroup Analyses by Surgical Complexity and Timing of Handover in the Inverse Probability of Treatment Weighted Cohort eTable 2. Exploratory Analysis by the Timing of Anesthesia Handover Relative to Cardiopulmonary Bypass in the Inverse Probability of Treatment Weighted Cohort eTable 3. Sensitivity Analysis Where 30-Day Mortality Was Modeled Using Multivariable Cox Proportional Hazard Regression in the Original Cohort eTable 4. Sensitivity Analysis Where 1-Year Mortality Was Modeled Using Multivariable Cox Proportional Hazard Regression in the Original Cohort eTable 5. Sensitivity Analysis Where 30-Day PACE Was Modeled Using Multivariable Cause-Specific Hazard Regression in the Original Cohort eTable 6. Sensitivity Analysis Where 1-Year PACE Was Modeled Using Multivariable Cause-Specific Hazard Regression in the Original Cohort eTable 7. Sensitivity Analysis Where ICU Length of Stay Was Modeled Using Multivariable Poisson Regression in the Original Cohort eTable 8. Sensitivity Analysis Where ICU Length of Stay Was Modeled Using Multivariable Negative Binomial Regression in the Original Cohort eTable 9. Sensitivity Analysis Where Hospital Length of Stay Was Modeled Using Multivariable Poisson Regression in the Original Cohort eTable 10. Sensitivity Analysis Where Hospital Length of Stay Was Modeled Using Multivariable Negative Binomial Regression in the Original Cohort eFigure. Cohort Flow Diagram [file jamanetwopen-e2148161-s001.pdf]

Supplemental Online Content

Sun LY, Jones PM, Wijeysundera DN, Mamas MA, Bader Eddeen A, O'Connor J. Association between handover of anesthesiology care and 1-year mortality among adults undergoing cardiac surgery. *JAMA Netw Open*. 2022;5(2):e2148161. doi:10.1001/jamanetworkopen.2021.48161

eTable 1. Subgroup Analyses by Surgical Complexity and Timing of Handover in the Inverse Probability of Treatment Weighted Cohort

eTable 2. Exploratory Analysis by the Timing of Anesthesia Handover Relative to Cardiopulmonary Bypass in the Inverse Probability of Treatment Weighted Cohort

eTable 3. Sensitivity Analysis Where 30-Day Mortality Was Modeled Using Multivariable Cox Proportional Hazard Regression in the Original Cohort

eTable 4. Sensitivity Analysis Where 1-Year Mortality Was Modeled Using Multivariable Cox Proportional Hazard Regression in the Original Cohort

eTable 5. Sensitivity Analysis Where 30-Day PACE Was Modeled Using Multivariable Cause-Specific Hazard Regression in the Original Cohort

eTable 6. Sensitivity Analysis Where 1-Year PACE Was Modeled Using Multivariable Cause-Specific Hazard Regression in the Original Cohort

eTable 7. Sensitivity Analysis Where ICU Length of Stay Was Modeled Using Multivariable Poisson Regression in the Original Cohort

eTable 8. Sensitivity Analysis Where ICU Length of Stay Was Modeled Using Multivariable Negative Binomial Regression in the Original Cohort

eTable 9. Sensitivity Analysis Where Hospital Length of Stay Was Modeled Using Multivariable Poisson Regression in the Original Cohort

eTable 10. Sensitivity Analysis Where Hospital Length of Stay Was Modeled Using Multivariable Negative Binomial Regression in the Original Cohort

eFigure. Cohort Flow Diagram

This supplemental material has been provided by the authors to give readers additional information about their work.

© 2022 Sun LY et al. *JAMA Network Open.*

eTable 1. Subgroup Analyses by Surgical Complexity and Timing of Handover in the Inverse Probability of Treatment Weighted Cohort

Outcome	Subgroup	Effect Measure [a] (95% CI)	*P*-value for interaction
**Complex vs. simple surgery**			
Primary outcome			
All-cause death within 30 d	Simple	1.69 (0.99-2.88)	0.47
	Complex	2.12 (1.53-2.94)	
All-cause death within 1 yr	Simple	1.31 (0.92-1.87)	0.03
	Complex	2.21 (1.61-3.02)	
Secondary outcomes			
PACE within 30 d	Simple	0.77 (0.51-1.18)	0.01
	Complex	1.85 (1.17-2.93)	
PACE within 1 yr	Simple	0.73 (0.54-0.99)	0.01
	Complex	1.37 (0.92-2.03)	
ICU length of stay	Simple	1.35 (1.17-1.54)	0.35
	Complex	1.61 (1.13-2.30)	
Hospital length of stay	Simple	1.10 (1.01-1.21)	0.09
	Complex	1.33 (1.09-1.62)	
**Evening, night or weekend vs. day-time case start**			
Primary outcome			
All-cause death within 30 d	Morning	2.14 (1.54-2.95)	0.0005
	Evening, night or WE	0.73 (0.44-1.21)	
All-cause death within 1 yr	Morning	1.65 (1.26-2.16)	0.5
	Evening, night or WE	1.35 (0.81-2.24)	
Secondary outcomes			
PACE within 30 d	Morning	0.98 (0.69-1.40)	0.24
	Evening, night or WE	1.56 (0.79-3.09)	
PACE within 1 yr	Morning	0.80 (0.60-1.05)	0.04
	Evening, night or WE or night	1.47 (0.89-2.41)	
ICU length of stay	Morning	1.35 (1.19-1.52)	0.54
	Evening, night or WE	1.66 (0.85-3.23)	
Hospital length of stay	Morning	1.12 (1.03-1.21)	0.28
	Evening, night or WE	1.39 (0.94-2.05)	

[a] Hazard ratios (HR) were provided for binary outcomes (death, PACE) and rate ratios (RR) were provided for continuous outcomes (ICU and hospital LOS)

Abbreviations: PACE = patient-relevant adverse cardiac and noncardiac events; ICU = intensive care unit; IQR = interquartile range; WE = weekend

© 2022 Sun LY et al. *JAMA Network Open.*

eTable 2. Exploratory Analysis by the Timing of Anesthesia Handover Relative to Cardiopulmonary Bypass in the Inverse Probability of Treatment Weighted Cohort

Outcome	Subgroup	Effect Measure [a] (95% CI)	*P*-value
Primary outcome			
All-cause death within 30 d	During or After Bypass	2.25 (1.45-3.5)	0.0003
	Before Bypass	1.73 (1.18-2.54)	0.005
	No Handover	Ref	
All-cause death within 1 yr	During or After Bypass	1.57 (1.07-2.31)	0.02
	Before Bypass	1.71 (1.26-2.31)	0.0005
	No Handover	Ref	
Secondary outcomes			
PACE within 30 d	During or After Bypass	1.77 (1.11-2.83)	0.02
	Before Bypass	0.78 (0.51-1.19)	0.26
	No Handover	Ref	
PACE within 1 yr	During or After Bypass	1.17 (0.79-1.75)	0.43
	Before Bypass	0.77 (0.57-1.04)	0.08
	No Handover	Ref	
ICU length of stay	During or After Bypass	1.29 (1.10-1.51)	0.002
	Before Bypass	1.50 (1.21-1.86)	0.0002
	No Handover	Ref	
Hospital length of stay	During or After Bypass	1.14 (1.02-1.27)	0.02
	Before Bypass	1.18 (1.04-1.33)	0.009
	No Handover	Ref	

[a] Hazard ratios (HR) were provided for binary outcomes (death, PACE) and rate ratios (RR) were provided for continuous outcomes (ICU and hospital LOS)

Abbreviations: PACE = patient-relevant adverse cardiac and noncardiac events; ICU = intensive care unit; IQR = interquartile range; WE = weekend

eTable 3. Sensitivity Analysis Where 30-Day Mortality Was Modeled Using Multivariable Cox Proportional Hazard Regression in the Original Cohort

Variable	Hazard Ratio (95% CI)	*P*-value
**Demographics**		
Age	1.03 (1.02-1.03)	<.0001
Male	0.75 (0.69-0.82)	<.0001
Body mass index	1.00 (0.99-1.01)	0.88
Rural residence,	0.96 (0.86-1.08)	0.54
Hospital type		
Community	1.21 (1.06-1.39)	0.01
Teaching		
Income quintile		
1	1.35 (1.18-1.55)	<.0001
2	1.19 (1.04-1.36)	0.01
3	1.21 (1.06-1.39)	0.01
4	1.16 (1.01-1.33)	0.04
5	Reference	Reference
**Comorbidities**		
Hypertension	1.14 (0.98-1.33)	0.08
Atrial fibrillation	1.23 (1.09-1.38)	0.0007
Recent MI	1.27 (1.13-1.42)	<.0001
CCS class		
0	Reference	Reference
1	0.88 (0.73-1.07)	0.20
2	0.94 (0.78-1.12)	0.46
3	1.02 (0.86-1.21)	0.79
4	1.64 (1.31-2.07)	<.0001
Low-risk ACS	1.20 (1.01-1.42)	0.04
Intermediate-risk ACS	1.32 (1.10-1.58)	0.003
High-risk ACS	1.81 (1.42-2.32)	<.0001
Emergent	2.93 (2.35-3.66)	<.0001
Peripheral arterial disease	1.19 (1.08-1.32)	0.0007
LVEF		
≥ 50%	Reference	Reference
35-49%	1.26 (1.13-1.40)	<.0001
20-35%	1.61 (1.41-1.83)	<.0001
< 20%	2.40 (1.94-2.98)	<.0001
NYHA class		
1		
2	0.80 (0.69-0.92)	0.003
3	0.98 (0.84-1.13)	0.75
4	1.23 (1.05-1.43)	0.01
Heart failure	1.64 (1.48-1.81)	<.0001
Endocarditis		
None	Reference	Reference
Active	1.84 (1.45-2.34)	<.0001
Subacute	0.54 (0.30-1.00)	0.05
Cerebrovascular disease	1.30 (1.16-1.45)	<.0001

© 2022 Sun LY et al. *JAMA Network Open.*

Variable	Hazard Ratio (95% CI)	*P*-value
Smoker		
Never	Reference	Reference
Current	1.07 (0.95-1.21)	0.29
Former	1.07 (0.97-1.18)	0.16
Diabetes	1.05 (0.95-1.15)	0.36
GFR	0.99 (0.98-0.99)	<.0001
Dialysis	1.13 (0.93-1.37)	0.21
Anemia	1.06 (0.95-1.18)	0.29
Liver disease	1.62 (1.21-2.16)	0.00
Dementia	1.21 (0.94-1.55)	0.14
Depression	0.97 (0.73-1.28)	0.82
Psychosis	1.10 (0.49-2.46)	0.82
Malignancy	0.92 (0.78-1.09)	0.33
**Operative characteristics**		
**Anesthesia Handover**	**1.50 (1.25-1.81)**	**<.0001**
Surgery type		
CABG	Reference	Reference
MultiValve	2.61 (2.04-3.34)	<.0001
SingleValve	1.65 (1.40-1.94)	<.0001
CABG + Single Valve	1.78 (1.55-2.05)	<.0001
CABG + Multi Valve	2.38 (1.81-3.14)	<.0001
Thoracic Aorta	3.56 (3.13-4.04)	<.0001
Redo sternotomy	1.31 (1.12-1.54)	0.0008
Cardiogenic shock	1.58 (1.22-2.04)	0.0006
Operative priority		
Emergent	1.24 (0.99-1.56)	0.06
Urgent	1.06 (0.92-1.23)	0.43
Semi-urgent	1.21 (1.05-1.38)	0.01
Elective	Reference	Reference
**Physician characteristics**		
Surgeon age	1.01 (1.00-1.01)	0.14
Female Surgeon	0.95 (0.81-1.12)	0.54
Surgeon volume	1.00 (1.00-1.00)	.
Anesthesiologist age	1.00 (0.99-1.01)	0.51
Female anesthesiologist	0.96 (0.84-1.09)	0.51
Anesthesiologist volume		
<500	1.06 (0.81-1.38)	0.66
500-999	1.17 (0.92-1.48)	0.21
1000-1999	1.06 (0.85-1.31)	0.61
≥2000	Reference	Reference
Surgery Duration		
<300 min	0.16 (0.14-0.18)	<.0001
300-479 min	0.31 (0.27-0.35)	<.0001
≥480 min	Reference	Reference

Abbreviations: GFR - Glomerular Filtration fraction, LVEF = Left Ventricle Ejection Fraction, MI = myocardial infarction; CCS = Canadian Cardiovascular Society; ACS = acute coronary syndrome; NYHA = New York Heart Association; CABG = coronary artery bypass grafting

© 2022 Sun LY et al. *JAMA Network Open.*

eTable 4. Sensitivity Analysis Where 1-Year Mortality Was Modeled Using Multivariable Cox Proportional Hazard Regression in the Original Cohort

Variable	Hazard Ratio (95% CI)	*P*-value
**Demographics**		
Age	1.036 (1.032-1.039)	<0.001
Male	0.85 (0.80-0.91)	<0.001
Body mass index	0.999 (0.994-1.003)	0.59
Rural residence,	0.98 (0.91-1.06)	0.67
Hospital type		
Community	1.12 (1.02-1.24)	0.02
Teaching		
Income quintile		
1	1.32 (1.20–1.44)	<0.001
2	1.17 (1.07-1.28)	<0.001
3	1.11 (1.01-1.21)	0.03
4	1.07 (0.97-1.17)	0.18
5	Reference	Reference
**Comorbidities**		
Hypertension	1.07 (0.96-1.18)	0.21
Atrial fibrillation	1.35 (1.25-1.46)	<.0001
Recent MI	1.11 (1.03-1.21)	0.01
CCS class		
0	Reference	Reference
1	0.97 (0.86-1.09)	0.57
2	0.89 (0.79-1.00)	0.05
3	1.04 (0.93-1.16)	0.52
4	1.44 (1.23-1.68)	<.0001
Low-risk ACS	1.22 (1.09-1.36)	0.004
Intermediate-risk ACS	1.29 (1.14-1.45)	<.0001
High-risk ACS	1.54 (1.29-1.84)	<.0001
Emergent	2.30 (1.95-2.72)	<.0001
Peripheral arterial disease	1.30 (1.21-1.39)	<.0001
LVEF		
≥ 50%	Reference	Reference
35-49%	1.27 (1.19-1.37)	<.0001
20-35%	1.48 (1.35-1.62)	<.0001
< 20%	1.90 (1.61-2.24)	<.0001
NYHA class		
1	0.98 (0.90-1.06)	0.63
2	0.87 (0.79-0.96)	0.004
3	1.00 (0.91-1.10)	0.97
4	1.29 (1.15-1.44)	<.0001
Heart failure	1.70 (1.59-1.82)	<.0001
Endocarditis		
None	Reference	Reference
Active	2.00 (1.70-2.36)	<.0001
Subacute	0.91 (0.64-1.29)	0.58
Cerebrovascular disease	1.25 (1.16-1.35)	<.0001

© 2022 Sun LY et al. *JAMA Network Open.*

Variable	Hazard Ratio (95% CI)	*P*-value
Smoker		
Never	Reference	Reference
Current	1.28 (1.18-1.39)	<.0001
Former	1.13 (1.06-1.20)	0.0003
Diabetes	1.22 (1.15-1.30)	<.0001
GFR	0.988 (0.986-0.989)	<.0001
Dialysis	1.29 (1.13-1.46)	<.0001
Anemia	1.26 (1.17-1.35)	<.0001
Liver disease	2.09 (1.77-2.47)	<.0001
Dementia	1.22 (1.03-1.44)	0.02
Depression	1.37 (1.16-1.61)	0.0002
Psychosis	1.04 (0.62-1.73)	0.88
Malignancy	1.54 (1.41-1.69)	<.0001
**Operative characteristics**		
**Anesthesia Handover**	**1.52 (1.3-1.76)**	**<.0001**
Surgery type		
CABG	Reference	Reference
MultiValve	2.22 (1.88-2.62)	<.0001
SingleValve	1.64 (1.47-1.82)	<.0001
CABG +Single Valve	1.61 (1.47-1.76)	<.0001
CABG + Multi Valve	1.92 (1.57-2.35)	<.0001
Thoracic Aorta	2.66 (2.42-2.91)	<.0001
Redo sternotomy	1.54 (1.25-1.90)	<.0001
Cardiogenic shock	1.17 (1.03-1.31)	0.01
Operative priority		
Emergent	1.14 (0.96-1.34)	0.13
Urgent	1.06 (0.96-1.16)	0.27
Semi-urgent	1.08 (0.99-1.18)	0.08
Elective	Reference	Reference
**Physician characteristics**		
Surgeon age	1.006 (1.001-1.012)	0.03
Female Surgeon	0.91 (0.81-1.01)	0.09
Surgeon volume	1 (1.00-1.00)	1.0
Anesthesiologist age	1.0 (0.995-1.0006)	0.95
Female anesthesiologist	0.98 (0.89-1.07)	0.59
Anesthesiologist volume		
<500	1.17 (0.97-1.40)	1.40
500-999	1.11 (0.94-1.30)	1.30
1000-1999	1.12 (0.97-1.30)	1.30
≥2000	Reference	Reference
Surgery Duration		
<300 min	0.28 (0.25-0.31)	<.0001
300-479 min	0.41 (0.37-0.45)	<.0001
≥480 min	Reference	Reference

Abbreviations: GFR - Glomerular Filtration fraction, LVEF = Left Ventricle Ejection Fraction, MI = myocardial infarction; CCS = Canadian Cardiovascular Society; ACS = acute coronary syndrome; NYHA = New York Heart Association; CABG = coronary artery bypass grafting

© 2022 Sun LY et al. *JAMA Network Open.*

eTable 5. Sensitivity Analysis Where 30-Day PACE Was Modeled Using Multivariable Cause-Specific Hazard Regression in the Original Cohort

Variable	Hazard Ratio (95% CI)	*P*-value
**Demographics**		
Age	1.03 (1.02-1.03)	<.0001
Male	0.82 (0.76-0.88)	<.0001
Body mass index	1.01 (1.01-1.01)	<.0001
Rural residence	0.96 (0.88-1.04)	0.31
Hospital type		
Community	1.09 (0.98-1.21)	0.10
Income quintile		
1	1.18 (1.07-1.31)	0.001
2	1.16 (1.05-1.28)	0.003
3	1.19 (1.08-1.32)	0.0004
4	1.03 (0.93-1.14)	0.52
5	Reference	Reference
**Comorbidities**		
Hypertension	1.16 (1.04-1.29)	0.01
Atrial fibrillation	1.16 (1.04-1.30)	0.01
Recent MI	1.13 (1.03-1.24)	0.01
CCS class		
0	Reference	Reference
1	1.00 (0.88-1.14)	0.9967
2	0.85 (0.75-0.95)	0.01
3	1.07 (0.95-1.20)	0.26
4	1.17 (0.97-1.41)	0.09
Low-risk ACS	1.07 (0.94-1.22)	0.31
Intermediate-risk ACS	1.12 (0.96-1.30)	0.15
High-risk ACS	1.20 (0.95-1.52)	0.12
Emergent	1.74 (1.39-2.18)	<.0001
Peripheral arterial disease	1.12 (1.03-1.22)	0.01
LVEF		
≥ 50%	Reference	Reference
35-49%	1.09 (1.01-1.19)	0.03
20-35%	1.26 (1.12-1.43)	0.00
< 20%	1.66 (1.30-2.12)	<.0001
NYHA class		
1	Reference	Reference
2	0.96 (0.87-1.07)	0.50
3	1.04 (0.92-1.17)	0.51
4	1.19 (1.00-1.41)	0.05
Heart failure	0.41 (0.38-0.45)	<.0001
Endocarditis		
None	Reference	Reference
Active	2.11 (1.65-2.70)	<.0001
Subacute	1.13 (0.67-1.88)	0.65
Cerebrovascular disease	1.24 (1.13-1.36)	<.0001
Smoker		
Never	Reference	Reference

© 2022 Sun LY et al. *JAMA Network Open.*

**Variable**	**Hazard Ratio (95% CI)**	***P*-value**
Current	1.09 (1.00-1.20)	0.06
Former	1.05 (0.98-1.13)	0.17
Diabetes	1.12 (1.04-1.20)	0.002
GFR	0.99 (0.99-0.99)	<.0001
Dialysis	0.97 (0.79-1.18)	0.74
Anemia	1.28 (1.17-1.41)	<.0001
Liver disease	1.33 (1.00-1.75)	0.05
Dementia	0.93 (0.73-1.18)	0.54
Depression	1.40 (1.12-1.74)	0.002
Psychosis	0.82 (0.39-1.74)	0.61
Malignancy	1.04 (0.91-1.18)	0.56
**Operative characteristics**		
**Anesthesia Handover**	**1.13 (0.92-1.39)**	**0.23**
Surgery type		
CABG	Reference	Reference
MultiValve	1.66 (1.30-2.11)	<.0001
SingleValve	1.43 (1.27-1.61)	<.0001
CABG + Single Valve	1.65 (1.48-1.83)	<.0001
CABG + Multi Valve	1.77 (1.30-2.40)	0.0002
Thoracic Aorta	1.89 (1.69-2.12)	<.0001
Redo sternotomy	0.92 (0.76-1.10)	0.35
Cardiogenic shock	1.11 (0.79-1.55)	0.54
Operative priority		
Emergent	1.15 (0.92-1.42)	0.21
Urgent	1.09 (0.98-1.22)	0.12
Semi-urgent	1.06 (0.98-1.16)	0.16
Elective	Reference	Reference
**Physician characteristics**		
Surgeon age	1.00 (0.99-1.01)	0.94
Female Surgeon	0.84 (0.74-0.94)	0.004
Surgeon volume	1.00 (1.00-1.00)	1.0
Anesthesiologist age	1.00 (1.00-1.01)	0.66
Female anesthesiologist	0.87 (0.79-0.97)	0.01
Anesthesiologist volume		
<500	1.28 (1.05-1.56)	0.02
500-999	1.16 (0.97-1.38)	0.11
1000-1999	1.10 (0.94-1.29)	0.23
≥2000	Reference	Reference
Surgery Duration		
<300 min	0.44 (0.38-0.52)	<.0001
300-479 min	0.52 (0.45-0.61)	<.0001
≥480 min	Reference	Reference

Abbreviations: GFR - Glomerular Filtration fraction, LVEF = Left Ventricle Ejection Fraction, MI = myocardial infarction; CCS = Canadian Cardiovascular Society; ACS = acute coronary syndrome; NYHA = New York Heart Association; CABG = coronary artery bypass grafting

© 2022 Sun LY et al. *JAMA Network Open.*

eTable 6. Sensitivity Analysis Where 1-Year PACE Was Modeled Using Multivariable Cause-Specific Hazard Regression in the Original Cohort

Variable	Hazard Ratio (95% CI)	*P*-value
**Demographics**		
Age	1.028 (1.025-1.031)	<.0001
Male	0.80 (0.76-0.84)	<.0001
Body mass index	1.01 (1.01-1.01)	<.0001
Rural residence	0.96 (0.90-1.02)	0.17
Hospital type		
Community	1.08 (0.98-1.18)	0.12
Income quintile		
1	1.20 (1.12-1.29)	<.0001
2	1.11 (1.04-1.19)	0.003
3	1.13 (1.05-1.21)	0.001
4	1.05 (0.97-1.12)	0.21
5	Reference	Reference
**Comorbidities**		
Hypertension	1.18 (1.09-1.27)	<.0001
Atrial fibrillation	1.27 (1.18-1.38)	<.0001
Recent MI	1.14 (1.06-1.21)	0.0002
CCS class		
0	Reference	Reference
1	0.96 (0.88-1.06)	0.41
2	0.84 (0.78-0.92)	<.0001
3	1.00 (0.92-1.09)	0.96
4	1.04 (0.90-1.19)	0.61
Low-risk ACS	1.05 (0.96-1.15)	0.29
Intermediate-risk ACS	1.11 (0.99-1.23)	0.06
High-risk ACS	1.19 (1.00-1.41)	0.04
Emergent	1.61 (1.36-1.91)	<.0001
Peripheral arterial disease	1.14 (1.08-1.21)	<.0001
LVEF		
≥ 50%	Reference	Reference
35-49%	1.28 (1.21-1.36)	<.0001
20-35%	1.67 (1.54-1.81)	<.0001
< 20%	1.96 (1.64-2.34)	<.0001
NYHA class		
1	Reference	Reference
2	1.01 (0.94-1.09)	0.78
3	1.12 (1.03-1.22)	0.01
4	1.12 (0.98-1.28)	0.09
Heart failure	0.34 (0.32-0.37)	<.0001
Endocarditis		
None	Reference	Reference
Active	2.21 (1.82-2.67)	<.0001
Subacute	1.18 (0.80-1.73)	0.40
Cerebrovascular disease	1.24 (1.17-1.33)	<.0001
Smoker		
Never	Reference	Reference

© 2022 Sun LY et al. *JAMA Network Open.*

Variable	Hazard Ratio (95% CI)	*P*-value
Current	1.16 (1.09-1.23)	<.0001
Former	1.05 (1.00-1.11)	0.05
Diabetes	1.22 (1.16-1.28)	<.0001
GFR	0.99 (0.99-0.99)	<.0001
Dialysis	1.01 (0.87-1.16)	0.94
Anemia	1.29 (1.20-1.37)	<.0001
Liver disease	1.33 (1.09-1.63)	0.01
Dementia	1.35 (1.16-1.56)	<.0001
Depression	1.38 (1.18-1.61)	<.0001
Psychosis	1.14 (0.73-1.77)	0.56
Malignancy	1.04 (0.95-1.14)	0.39
**Operative characteristics**		
**Anesthesia Handover**	1.00 (0.85-1.18)	**0.99**
Surgery type		
CABG	Reference	Reference
MultiValve	1.46 (1.22-1.76)	<.0001
SingleValve	1.27 (1.17-1.39)	<.0001
CABG + Single Valve	1.47 (1.36-1.59)	<.0001
CABG + Multi Valve	1.47 (1.16-1.86)	0.001
Thoracic Aorta	1.55 (1.42-1.69)	<.0001
Redo sternotomy	0.91 (0.79-1.04)	0.17
Cardiogenic shock	1.18 (0.92-1.51)	0.18
Operative priority		
Emergent	1.14 (0.97-1.34)	0.11
Urgent	1.09 (1.01-1.18)	0.03
Semi-urgent	1.05 (0.99-1.12)	0.13
Elective	Reference	Reference
**Physician characteristics**		
Surgeon age	1.00 (1.00-1.01)	0.12
Female Surgeon	0.87 (0.80-0.95)	0.002
Surgeon volume	1.00 (1.00-1.00)	.
Anesthesiologist age	1.00 (1.00-1.01)	0.68
Female anesthesiologist	0.86 (0.79-0.95)	0.002
Anesthesiologist volume		
<500	1.22 (1.04-1.43)	0.02
500-999	1.12 (0.98-1.30)	0.10
1000-1999	1.09 (0.96-1.23)	0.17
≥2000	Reference	Reference
Surgery Duration		
<300 min	0.51 (0.45-0.58)	<.0001
300-479 min	0.60 (0.53-0.68)	<.0001
≥480 min	Reference	Reference

Abbreviations: GFR - Glomerular Filtration fraction, LVEF = Left Ventricle Ejection Fraction, MI = myocardial infarction; CCS = Canadian Cardiovascular Society; ACS = acute coronary syndrome; NYHA = New York Heart Association; CABG = coronary artery bypass grafting

© 2022 Sun LY et al. *JAMA Network Open.*

eTable 7. Sensitivity Analysis Where ICU Length of Stay Was Modeled Using Multivariable Poisson Regression in the Original Cohort

Variable	Risk Ratio (95% CI)	*P*-value
**Demographics**		
Age	1.01 (1.01-1.012)	<.0001
Male	0.97 (0.94-1.00)	0.03
Body mass index	1 (1.002-1.005)	<.0001
Rural residence	0.99 (0.95-1.03)	0.60
Hospital type		
Community	0.97 (0.93-1.02)	0.21
Income quintile		
1	1.15 (1.10-1.20)	<.0001
2	1.09 (1.05-1.13)	<.0001
3	1.07 (1.03-1.11)	0.0003
4	1.03 (0.99-1.07)	0.12
5	Reference	Reference
**Comorbidities**		
Hypertension	1.01 (0.97-1.04)	0.68
Atrial fibrillation	1.14 (1.07-1.21)	<.0001
Recent MI	1.03 (0.99-1.07)	0.12
CCS class		
0	Reference	Reference
1	1.01 (0.95-1.07)	0.69
2	0.97 (0.92-1.02)	0.20
3	1.01 (0.96-1.06)	0.71
4	1.19 (1.05-1.34)	0.01
Low-risk ACS	1.01 (0.96-1.07)	0.69
Intermediate-risk ACS	1.11 (1.03-1.18)	0.004
High-risk ACS	1.27 (1.14-1.42)	<.0001
Emergent	1.74 (1.53-1.98)	<.0001
Peripheral arterial disease	1.05 (1.01-1.10)	0.02
LVEF		
≥ 50%	Reference	Reference
35-49%	1.06 (1.02-1.10)	0.001
20-35%	1.15 (1.10-1.21)	<.0001
< 20%	1.31 (1.20-1.42)	<.0001
NYHA class		
1	0.98 (0.90-1.06)	0.63
2	0.99 (0.94-1.03)	0.54
3	1.05 (1.00-1.11)	0.06
4	1.16 (1.07-1.26)	0.0005
Heart failure	1.08 (1.04-1.12)	<.0001
Endocarditis		
None	Reference	Reference
Active	1.34 (1.19-1.51)	<.0001
Subacute	0.89 (0.75-1.06)	0.20
Cerebrovascular disease	1.04 (1.00-1.09)	0.07
Smoker		
Never	Reference	Reference

© 2022 Sun LY et al. *JAMA Network Open.*

Variable	Risk Ratio (95% CI)	*P*-value
Current	1.05 (1.01-1.09)	0.01
Former	1 (0.97-1.03)	0.90
Diabetes	1.06 (1.03-1.09)	0.0001
GFR	1 (0.994-0.996)	<.0001
Dialysis	1.24 (1.10-1.40)	0.0004
Anemia	1.31 (1.25-1.39)	<.0001
Liver disease	1.1 (0.98-1.24)	0.10
Dementia	1.1 (1.00-1.21)	0.06
Depression	1.39 (1.22-1.60)	<.0001
Psychosis	0.97 (0.78-1.19)	0.75
Malignancy	1.02 (0.97-1.07)	0.53
**Operative characteristics**		
**Anesthesia Handover**	**1.27 (1.17-1.37)**	**<.0001**
Surgery type		
CABG	Reference	Reference
MultiValve	1.58 (1.44-1.74)	<.0001
SingleValve	1.27 (1.21-1.34)	<.0001
CABG + Single Valve	1.36 (1.29-1.44)	<.0001
CABG + Multi Valve	1.7 (1.48-1.95)	<.0001
Thoracic Aorta	1.7 (1.60-1.82)	<.0001
Redo sternotomy	0.99 (0.90-1.09)	0.84
Cardiogenic shock	1.19 (1.01-1.39)	0.03
Operative priority		
Emergent	1.05 (0.95-1.16)	0.33
Urgent	0.98 (0.94-1.03)	0.49
Semi-urgent	0.99 (0.96-1.03)	0.76
Elective	Reference	Reference
**Physician characteristics**		
Surgeon age	1 (0.99-1.0048)	0.45
Female Surgeon	0.96 (0.92-1.00)	0.03
Surgeon volume	1 (1.00-1.0000)	0.07
Anesthesiologist age	1 (0.99-1.0046)	0.29
Female anesthesiologist	1.01 (0.96-1.06)	0.72
Anesthesiologist volume		
<500	1.16 (1.06-1.26)	0.0007
500-999	1.17 (1.08-1.26)	0.0001
1000-1999	1.16 (1.09-1.23)	<.0001
≥2000	Reference	Reference
Surgery Duration		
<300 min	0.46 (0.42-0.50)	<.0001
300-479 min	0.62 (0.56-0.67)	<.0001
≥480 min	Reference	Reference

Abbreviations: GFR - Glomerular Filtration fraction, LVEF = Left Ventricle Ejection Fraction, MI = myocardial infarction; CCS = Canadian Cardiovascular Society; ACS = acute coronary syndrome; NYHA = New York Heart Association; CABG = coronary artery bypass grafting

© 2022 Sun LY et al. *JAMA Network Open.*

eTable 8. Sensitivity Analysis Where ICU Length of Stay Was Modeled Using Multivariable Negative Binomial Regression in the Original Cohort

Variable	Risk Ratio (95% CI)	*P*-value
**Demographics**		
Age	1.01 (1.01-1.01)	<.0001
Male	0.95 (0.93-0.98)	0.00
Body mass index	1.00 (1.00-1.00)	<.0001
Rural residence	1.00 (0.97-1.03)	0.94
Hospital type		
Community	0.96 (0.92-1.00)	0.05
Income quintile		
1	1.13 (1.09-1.18)	<.0001
2	1.09 (1.06-1.13)	<.0001
3	1.06 (1.03-1.10)	0.00
4	1.03 (1.00-1.07)	0.05
5	Reference	Reference
**Comorbidities**		
Hypertension	1.01 (0.98-1.04)	0.55
Atrial fibrillation	1.15 (1.09-1.20)	<.0001
Recent MI	1.04 (1.01-1.08)	0.02
CCS class		
0	Reference	Reference
1	1.01 (0.97-1.06)	0.58
2	0.99 (0.95-1.03)	0.56
3	1.01 (0.97-1.06)	0.62
4	1.13 (1.04-1.22)	0.002
Low-risk ACS	1.03 (0.99-1.08)	0.18
Intermediate-risk ACS	1.09 (1.04-1.15)	0.0013
High-risk ACS	1.24 (1.13-1.37)	<.0001
Emergent	1.78 (1.59-1.99)	<.0001
Peripheral arterial disease	1.06 (1.02-1.10)	0.002
LVEF		
≥ 50%	Reference	Reference
35-49%	1.06 (1.03-1.09)	<.0001
20-35%	1.16 (1.11-1.21)	<.0001
< 20%	1.31 (1.21-1.42)	<.0001
NYHA class		
1		
2	1.00 (0.97-1.04)	0.87
3	1.07 (1.02-1.12)	0.00
4	1.22 (1.14-1.32)	<.0001
Heart failure	1.27 (1.24-1.31)	<.0001
Endocarditis		
None	Reference	Reference
Active	1.47 (1.32-1.63)	<.0001
Subacute	0.90 (0.78-1.04)	0.17
Cerebrovascular disease	1.06 (1.02-1.09)	0.004
Smoker		
Never	Reference	Reference

© 2022 Sun LY et al. *JAMA Network Open.*

Variable	Risk Ratio (95% CI)	*P*-value
Current	1.07 (1.04-1.10)	<.0001
Former	1.01 (0.98-1.03)	0.49
Diabetes	1.05 (1.03-1.08)	<.0001
GFR	0.99 (0.99-1.00)	<.0001
Dialysis	1.22 (1.11-1.35)	<.0001
Anemia	1.30 (1.24-1.37)	<.0001
Liver disease	1.18 (1.07-1.31)	0.00
Dementia	1.16 (1.06-1.26)	0.00
Depression	1.39 (1.25-1.54)	<.0001
Psychosis	1.08 (0.88-1.33)	0.47
Malignancy	1.02 (0.97-1.07)	0.40
**Operative characteristics**		
**Anesthesia Handover**	**1.32 (1.22-1.41)**	**<.0001**
Surgery type		
CABG	Reference	Reference
MultiValve	1.58 (1.45-1.71)	<.0001
SingleValve	1.23 (1.18-1.28)	<.0001
CABG + Single Valve	1.35 (1.29-1.42)	<.0001
CABG + Multi Valve	1.73 (1.52-1.97)	<.0001
Thoracic Aorta	1.68 (1.58-1.78)	<.0001
Redo sternotomy	1.02 (0.94-1.11)	0.61
Cardiogenic shock	1.31 (1.13-1.52)	0.0004
Operative priority		
Emergent	1.07 (0.98-1.17)	0.13
Urgent	0.97 (0.93-1.01)	0.13
Semi-urgent	0.98 (0.96-1.01)	0.20
Elective	Reference	Reference
**Physician characteristics**		
Surgeon age	1.00 (1.00-1.00)	0.47
Female Surgeon	0.97 (0.93-1.01)	0.11
Surgeon volume	1.00 (1.00-1.00)	0.06
Anesthesiologist age	1.00 (1.00-1.00)	0.17
Female anesthesiologist	1.01 (0.96-1.07)	0.60
Anesthesiologist volume		
<500	1.15 (1.06-1.25)	0.0007
500-999	1.16 (1.07-1.25)	0.0001
1000-1999	1.14 (1.07-1.22)	<.0001
≥2000	Reference	Reference
Surgery Duration		
<300 min	0.44 (0.41-0.48)	<.0001
300-479 min	0.59 (0.54-0.64)	<.0001
≥480 min	Reference	Reference

Abbreviations: GFR - Glomerular Filtration fraction, LVEF = Left Ventricle Ejection Fraction, MI = myocardial infarction; CCS = Canadian Cardiovascular Society; ACS = acute coronary syndrome; NYHA = New York Heart Association; CABG = coronary artery bypass grafting

© 2022 Sun LY et al. *JAMA Network Open.*

eTable 9. Sensitivity Analysis Where Hospital Length of Stay Was Modeled Using Multivariable Poisson Regression in the Original Cohort

Variable	Risk Ratio (95% CI)	*P*-value
**Demographics**		
Age	1.01 (1.01-1.01)	<.0001
Male	0.94 (0.93-0.96)	<.0001
Body mass index	1.003 (1.002-1.004)	<.0001
Rural residence	0.94 (0.92-0.96)	<.0001
Hospital type		
Community	1.03 (1.01-1.05)	0.01
Income quintile		
1	1.11 (1.08-1.13)	<.0001
2	1.07 (1.05-1.09)	<.0001
3	1.05 (1.03-1.07)	<.0001
4	1.03 (1.01-1.05)	0.01
5	Reference	Reference
**Comorbidities**		
Hypertension	1.02 (1.00-1.04)	0.10
Atrial fibrillation	1.08 (1.04-1.11)	<.0001
Recent MI	1.00 (0.97-1.02)	0.67
CCS class		
0	Reference	Reference
1	0.99 (0.96-1.02)	0.49
2	0.95 (0.93-0.98)	0.00
3	0.99 (0.97-1.02)	0.49
4	1.07 (1.02-1.12)	0.01
Low-risk ACS	1.00 (0.97-1.02)	0.80
Intermediate-risk ACS	1.08 (1.04-1.12)	<.0001
High-risk ACS	1.13 (1.06-1.20)	<.0001
Emergent	1.41 (1.30-1.53)	<.0001
Peripheral arterial disease	1.04 (1.02-1.07)	0.003
LVEF		
≥ 50%	Reference	Reference
35-49%	1.03 (1.01-1.04)	0.004
20-35%	1.06 (1.03-1.10)	<.0001
< 20%	1.17 (1.10-1.24)	<.0001
NYHA class		
1		
2	0.98 (0.96-1.00)	0.04
3	1.03 (1.01-1.06)	0.02
4	1.09 (1.04-1.15)	0.0006
Heart failure	1.19 (1.17-1.21)	<.0001
Endocarditis		
None	Reference	Reference
Active	1.42 (1.31-1.54)	<.0001
Subacute	1.04 (0.93-1.17)	0.47
Cerebrovascular disease	1.06 (1.04-1.09)	<.0001
Smoker		
Never	Reference	Reference

© 2022 Sun LY et al. *JAMA Network Open.*

Variable	Risk Ratio (95% CI)	*P*-value
Current	1.03 (1.00-1.05)	0.03
Former	0.98 (0.96-1.00)	0.01
Diabetes	0.997 (0.9966-0.9974)	<.0001
GFR	1.18 (1.11-1.27)	<.0001
Dialysis	1.24 (1.20-1.28)	<.0001
Anemia	1.07 (0.99-1.16)	0.07
Liver disease	1.19 (1.12-1.27)	<.0001
Dementia	1.38 (1.29-1.47)	<.0001
Depression	1.14 (1.01-1.29)	0.03
Psychosis	1.02 (0.99-1.05)	0.22
Malignancy	1.00 (1.00-1.00)	<.0001
**Operative characteristics**		
**Anesthesia Handover**	**1.15 (1.08-1.23)**	**<.0001**
Surgery type		
CABG	Reference	Reference
MultiValve	1.37 (1.30-1.44)	<.0001
SingleValve	1.16 (1.13-1.20)	<.0001
CABG + Single Valve	1.23 (1.20-1.27)	<.0001
CABG + Multi Valve	1.39 (1.27-1.51)	<.0001
Thoracic Aorta	1.34 (1.29-1.38)	<.0001
Redo sternotomy	0.95 (0.90-0.99)	0.03
Cardiogenic shock	1.02 (0.91-1.14)	0.75
Operative priority		
Emergent	1.04 (0.98-1.11)	0.17
Urgent	1.00 (0.98-1.03)	0.93
Semi-urgent	1.01 (0.99-1.03)	0.22
Elective	Reference	Reference
**Physician characteristics**		
Surgeon age	1.00 (1.00-1.00)	0.02
Female Surgeon	0.91 (0.89-0.94)	<.0001
Surgeon volume	1.00 (1.00-1.00)	0.02
Anesthesiologist age	1.00 (1.00-1.00)	0.04
Female anesthesiologist	1.02 (0.98-1.05)	0.34
Anesthesiologist volume		
<500	0.99 (0.95-1.03)	0.63
500-999	1.01 (0.97-1.06)	0.51
1000-1999	1.05 (1.01-1.09)	0.01
≥2000	Reference	Reference
Surgery Duration		
<300 min	0.60 (0.57-0.64)	<.0001
300-479 min	0.72 (0.68-0.76)	<.0001
≥480 min	Reference	Reference

Abbreviations: GFR - Glomerular Filtration fraction, LVEF = Left Ventricle Ejection Fraction, MI = myocardial infarction; CCS = Canadian Cardiovascular Society; ACS = acute coronary syndrome; NYHA = New York Heart Association; CABG = coronary artery bypass grafting

© 2022 Sun LY et al. *JAMA Network Open.*

eTable 10. Sensitivity Analysis Where Hospital Length of Stay Was Modeled Using Multivariable Negative Binomial Regression in the Original Cohort

Variable	Risk Ratio (95% CI)	*P*-value
**Demographics**		
Age	1.01 (1.009-1.011)	<.0001
Male	0.93 (0.92-0.95)	<.0001
Body mass index	1.003 (1.002-1.004)	<.0001
Rural residence	0.94 (0.92-0.96)	<.0001
Hospital type		
Community	1.03 (1.01-1.05)	0.0142
Income quintile		
1	1.10 (1.08-1.12)	<.0001
2	1.07 (1.06-1.09)	<.0001
3	1.05 (1.03-1.06)	<.0001
4	1.03 (1.01-1.05)	0.0006
5	Reference	Reference
**Comorbidities**		
Hypertension	1.02 (1.00-1.03)	0.0386
Atrial fibrillation	1.08 (1.06-1.11)	<.0001
Recent MI	1.00 (0.98-1.02)	0.9027
CCS class		
0	Reference	Reference
1	1.00 (0.97-1.02)	0.68
2	0.96 (0.94-0.98)	0.0005
3	0.99 (0.97-1.02)	0.59
4	1.06 (1.02-1.10)	0.002
Low-risk ACS	1.01 (0.98-1.03)	0.63
Intermediate-risk ACS	1.08 (1.04-1.11)	<.0001
High-risk ACS	1.42 (1.32-1.53)	<.0001
Emergent	1.12 (1.06-1.18)	<.0001
Peripheral arterial disease	1.05 (1.03-1.07)	<.0001
LVEF		
≥ 50%	Reference	Reference
35-49%	1.02 (1.01-1.04)	0.0014
20-35%	1.07 (1.04-1.09)	<.0001
< 20%	1.17 (1.10-1.24)	<.0001
NYHA class		
1	Reference	Reference
2	0.98 (0.97-1.00)	0.0602
3	1.04 (1.01-1.06)	0.0014
4	1.12 (1.07-1.17)	<.0001
Heart failure	1.18 (1.16-1.20)	<.0001
Endocarditis		
None	Reference	Reference
Active	1.51 (1.42-1.61)	<.0001
Subacute	1.04 (0.95-1.14)	0.36
Cerebrovascular disease	1.07 (1.05-1.09)	<.0001
Smoker		
Never	Reference	Reference

© 2022 Sun LY et al. *JAMA Network Open.*

**Variable**	**Risk Ratio (95% CI)**	***P*-value**
Current	1.51 (1.42-1.61)	<.0001
Former	1.04 (0.95-1.14)	0.36
Diabetes	1.06 (1.04-1.07)	<.0001
GFR	1.00 (1.00-1.00)	<.0001
Dialysis	1.18 (1.12-1.26)	<.0001
Anemia	1.24 (1.20-1.27)	<.0001
Liver disease	1.11 (1.04-1.19)	0.0016
Dementia	1.22 (1.15-1.30)	<.0001
Depression	1.37 (1.29-1.46)	<.0001
Psychosis	1.21 (1.07-1.37)	0.002
Malignancy	1.01 (0.99-1.04)	0.25
**Operative characteristics**		
**Anesthesia Handover**	**1.16 (1.09-1.23)**	**<.0001**
Surgery type		
CABG	Reference	Reference
MultiValve	1.38 (1.32-1.45)	<.0001
SingleValve	1.15 (1.13-1.18)	<.0001
CABG + Single Valve	1.23 (1.20-1.26)	<.0001
CABG + Multi Valve	1.40 (1.30-1.51)	<.0001
Thoracic Aorta	1.33 (1.29-1.38)	<.0001
Redo sternotomy	0.97 (0.92-1.01)	0.1659
Cardiogenic shock	1.08 (0.97-1.19)	0.1548
Operative priority		
Emergent	1.05 (0.99-1.10)	0.10
Urgent	1.00 (0.98-1.02)	0.88
Semi-urgent	1.01 (0.99-1.02)	0.38
Elective	Reference	Reference
**Physician characteristics**		
Surgeon age	1.002 (1.0007-1.0036)	0.004
Female Surgeon	0.92 (0.89-0.94)	<.0001
Surgeon volume	1.00 (1.00-1.00)	0.02
Anesthesiologist age	0.999 (0.997-0.999)	0.026
Female anesthesiologist	1.02 (0.99-1.05)	0.20
Anesthesiologist volume		
<500	0.98 (0.94-1.02)	0.22
500-999	1.00 (0.96-1.04)	0.97
1000-1999	1.04 (1.00-1.07)	0.03
≥2000	Reference	Reference
Surgery Duration		
<300 min	0.60 (0.57-0.63)	<.0001
300-479 min	0.71 (0.68-0.75)	<.0001
≥480 min	Reference	Reference

Abbreviations: GFR - Glomerular Filtration fraction, LVEF = Left Ventricle Ejection Fraction, MI = myocardial infarction; CCS = Canadian Cardiovascular Society; ACS = acute coronary syndrome; NYHA = New York Heart Association; CABG = coronary artery bypass grafting

© 2022 Sun LY et al. *JAMA Network Open.*

eFigure. Cohort Flow Diagram.

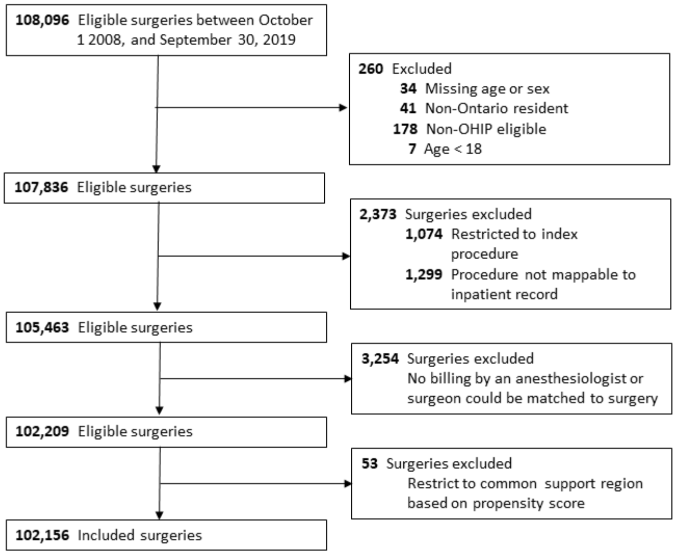

© 2022 Sun LY et al. *JAMA Network Open.*
